# When More Than Exercise Is Needed to Increase Chances of Aging in Place: Qualitative Analysis of a Telehealth Physical Activity Program to Improve Mobility in Low-Income Older Adults

**DOI:** 10.2196/11955

**Published:** 2018-12-21

**Authors:** Kathy VanRavenstein, Boyd H Davis

**Affiliations:** 1 College of Nursing Medical University of South Carolina Charleston, SC United States; 2 Applied Linguistics Department of English University North Carolina Charlotte Charlotte, NC United States

**Keywords:** older adults, low income, physical activity, aging in place, social isolation, qualitative research

## Abstract

**Background:**

A telehealth-delivered physical activity program was implemented within two low-income older adult housing properties utilizing the Otago exercise program, a physical therapy program endorsed by the Centers for Disease Control and Prevention to improve balance and strengthening in community dwelling older adults and by the National Council on Aging as the highest level of evidence for fall prevention programs. Participants were also given Fitbit activity monitors to help track their activity.

**Objective:**

The goal of this project was to increase older adults’ daily physical activity in hopes of decreasing chronic disease morbidity, disability, and falls, and decrease social isolation.

**Methods:**

The Otago exercise program was conducted via telehealth twice weekly for 12 weeks. Participants also wore Fitbit activity trackers to encourage physical activity outside of the group classes. Postintervention qualitative interviews were conducted, recorded, transcribed, and analyzed using discourse analysis.

**Results:**

Twenty-one older adult participants from two low-income properties in Charleston, SC, participated in the 12-week telehealth physical therapy program. Postintervention qualitative interviews revealed that the two sites were very different in their participation in the program and their main concerns surrounding aging in place. One site had a community-oriented outlook and enjoyed participating in physical activity together; whereas, the other site had very few participants and referenced depression and social isolation as main concerns.

**Conclusions:**

A telehealth physical therapy-led intervention to increase physical activity in low-income older adults aging in place was successfully implemented and attended; however, it became clear in postintervention qualitative interviews that social isolation and depression were prevalent and mental health needs to be addressed along with physical health to encourage successful aging in place.

## Introduction

Physical inactivity increases the risk of adverse health conditions in older adults, including coronary heart disease, type 2 diabetes, and breast and colon cancers [1]. Globally, 67% of adults older than 60 years are sedentary for more than 8.5 hours per day [2]. The World Health Organization calls for a 10% increase in physical activity (PA) and encourages community-based collaborations to formulate evidence-based, cost-effective means to achieve this goal [3]. PA can decrease the chance of falls [4], cardiovascular disease [5], and arthritis pain [6-8]. In addition, PA can increase memory performance [9] and improve functioning with arthritis [6-8]. Older adults spending less time in sedentary behavior have a greater chance to age successfully and to age in place [10], with improved health-related quality of life [9].

Incidence of chronic diseases, such as diabetes, cancer, heart disease, stroke, and Alzheimer disease, increase as people age. Three out of four adults in the United States have more than one chronic disease [11]. Chronic disease can greatly affect an individual’s ability to age in place by decreasing the ability to perform activities of daily living such as bathing, dressing, cooking, and taking medications. The inability to perform these activities can also restrict a person’s desire to interact with others, creating or increasing social isolation [12]. In turn, social isolation can lead to depression [13-15], which can also decrease seniors’ ability to care for themselves [16,17] and affects their ability to age in place. Social isolation in the older adult has also been linked to increased falls [18]. In this paper, we report on the first year of our project to increase low-income older adults’ daily PA in hopes of decreasing chronic disease morbidities, disability, and falls.

To address issues of aging in place in their low-income older adult residents, the Humanities Foundation, a nonprofit affordable housing provider in South Carolina, Georgia, Virginia, and Louisiana, partnered with the College of Nursing at the Medical University of South Carolina (MUSC). To better understand residents’ views on aging in place in their current living situation at Humanities Foundation properties, researchers from the MUSC had previously conducted a survey of interest in health care with 165 residents of Garden Vistas and five other low-income residential sites sponsored by the Humanities Foundation. In addition, nursing students conducted 14 individual interviews with residents at Garden Vistas, on their perceptions of aging in place. Predominant themes in those initial responses were fear and worry about personal safety, their ability to retain some degree of mobility, and social isolation [19].

Given current research on the intersections between low income, isolation, and depression, as well as other mental health issues, those initial responses and the 2017 interview replies are not surprising. For example, an earlier research review by Golant [20] noted that “an emerging literature casts doubt on whether staying put can be a one-size-fits-all solution.” Shin et al [21] noted the prevalence of depression for residents of public housing; Xiang et al [22] identified this depression as stemming from unmet needs. A National Council on Aging survey [23] highlighted the vulnerability of low-income adults; Brown et al [24] reported that nearly 40% of homeless adults had depression. A report by the Kenan Institute [25] identified renters as one of the three vulnerable groups who would need a great deal of help if they were to age in place.

Fitbit wearable fitness trackers meet high reliability and validity requirements for parameters such as step counts. Telehealth and remote videoconferencing have been effectively used to deliver rehabilitation after total knee replacement, strokes, and physical therapy to Veterans. Our project used wearable fitness trackers, combined with integrated feedback software and televideo to bring physical therapy (PT) student providers “into the room” with the older adult, hoping to provide motivation for the participants to become more physically active, and ultimately improving health, reducing falls and enhancing aging in place.

PA interventions incorporating technology have garnered research support for nursing and rehabilitation with older adults. Weinstock et al [4] utilized videoconferencing between diabetes educators and older adults to enhance pedometer effectiveness and found that participants in the telemedicine group had fewer declines in PA and physical impairment compared to those not using telemedicine. Silveira et al [5] and Wu et al [6] delivered PA interventions to older adults and found that community-based PA and videoconferencing produced higher retention, greater reduction in falls, and improvement in balance and health measures [5,6]. Compernolle et al [7] integrated pedometers and an internet-based PA program for adults at risk for chronic medical problems and found step counts and a computer-based intervention led to more accurate PA reports [7].

Activity trackers can increase PA when used in the appropriate context and are particularly effective with support infrastructure. Activity trackers providing real-time feedback are even more effective [26]. Fitbit wearable fitness trackers meet high reliability and validity requirements for parameters such as step counts [27]. Telehealth and remote videoconferencing have been effectively used to deliver rehabilitation after total knee replacement [28], stroke [29], and PT to Veterans [30].

Based on the information collected from residents of the Humanities Foundation, a telehealth-delivered PA program was implemented within two of the Humanities properties. It used the Otago exercise program, a PT program endorsed by the Centers for Disease Control and Prevention to improve balance and strengthening in community dwelling older adults [8] and by the National Council on Aging as the highest level of evidence for fall prevention programs [31]. Participants were also given wearable activity monitors (Fitbits) to help them track their own activity. The goal of the project was to increase older adults’ daily PA in hopes of decreasing chronic disease morbidities, disability, and falls. Combining the PA program with the activity tracker was intended to accustom both nursing and rehabilitation professionals to the combination of telehealth delivery and wearable technology as a means of reaching low-income seniors with varying mental health and typically sedentary lifestyle.

## Methods

### Sample Recruitment

The first sites chosen in collaboration with the Humanities Foundation were (1) Garden Vistas, two residences for seniors built since 2010, with 72 and 59 apartments respectively, located on the same road in a newly developing suburban area, and (2) Garden North, a 36-apartment cluster in the middle of the city. Each site offered one- and two-bedroom residences. After study approval was obtained from the MUSC institutional review board, flyers were sent to individual apartment mailboxes and posted in elevators and on bulletin boards. They advertised the free exercise class to be offered by MUSC and the opportunity to wear Fitbit fitness trackers. An on-site recruiting meeting was publicized and held. Inclusion criteria for the study were (1) older adults aged 55 years and older living in a Humanities Foundation apartment complex, (2) able to stand for 15 minutes, (3) able to walk 150 feet with or without an assistive device, (4) able to follow simple instructions for exercise, and (5) able to wear, operate, and charge the Fitbit HR fitness tracker. The following were exclusion criteria: (1) inability or unwillingness of participant to give informed consent and (2) physical, cognitive, sensory, or psychiatric disability that would limit participants from partaking in a PA program or wearing a fitness tracker.

### Physical Activity Program

Participants who met the inclusion criteria were entered into the study. Prior to the start of the 12-week program, participants wore the Fitbit HR fitness trackers for one week to record preintervention step counts. Participants were trained on the use of the fitness trackers and instructed to wear them daily. Data from the trackers was downloaded and shared with each participant on a weekly basis. Preintervention data were also collected on each participant using the Self-Efficacy for Exercise Scale [32], a demographic questionnaire, a 30-second sit-to-stand test [33], the Mini Balance Evaluation Systems Test, the Berg Balance Scale [34], and a two-minute walk test. This information was also collected postintervention. Participants then took part in a group telehealth PA using the Otago exercise program. The program was delivered to each site twice weekly via televideo by a PT student who was overseen by PT faculty from MUSC.

### Qualitative Interviews

Participants who had agreed to be in the program were told by the study coordinator that they would be telephoned by the second author, a professor of linguistics with more than 30 years’ experience in conducting and interpreting oral interviews. She introduced herself and reminded participants she was part of the team because of her research on language and aging and previewed the major questions she would ask (italicized in Textbox 1).

Calls were made to their residence using Skype; audio-recorded using Call Recorder; reformatted as wav files using Audacity; transcribed by VerbalInk, a HIPAA-compliant medical transcription company; and retained by the authors. All participants were called up to five times or until they answered; four never answered. Approximately 80% were reached and recorded. Seven (33%) were recorded twice; none refused the calls. Questions used were designed by the authors and reviewed by all team members (see Textbox 1). Interviews averaged 8 minutes. Follow-up interviews with Garden North participants averaging 5 minutes were collected a year later using the same interviewer, technique, equipment, and approach. In addition to asking if participants still exercised in any way, retained or used their Fitbits, and would be willing to join another exercise group, these interviews also included the Patient Health Questionnaire-2. There were 10 of 14 (71%) reached and recorded; one had moved away and the other three never answered.

**Preintervention and postintervention interview questions. Questions in italics are original interview questions. Nonitalicized questions are additional questions asked by interviewer. PT: physical therapy.**

**Preintervention Interview Questions**

1. *Tell me about your usual exercise/physical activity.* Do you usually exercise on your own? Do you ever volunteer with other programs or to help others? Tell me about that.

2. *Why do you think being physically active is important?*

3. *How do you feel about participating in group exercises?*

4. *How do you feel about participating in an exercise program over the TV?*

5. *Tell me what you know about Fitbits [tracker].* Have you ever worn one or any other type of activity bracelet? Would you be open to wearing one? What makes you think that you would be comfortable wearing one? What makes you think it would help increase your physical activity?

**Postintervention Interview Questions**

1. *Tell me about your current usual exercise/physical activity.* Do you usually exercise on your own? Do you ever volunteer with other programs or to help others? Tell me about that.

2. *Why do you think being physically active is important?*

3. *How do you feel about participating in group exercise?*

4. *What did you think about the physical activity program that was done over the TV?* What specific aspect did you like? Was there something specific that you disliked? What would you like to see done differently?

5. How did the nursing students do when they collected your vital signs, helped you with the Fitbit [tracker], and helped with the exercises? What do you think they could have done differently?

6. *How did the PT students do in giving the exercise program? What do you think they have done differently?*

7. *What did you think of the Fitbit?* Was it easy to use? How did it increase your physical activity—or did it? Why do you think many people like to wear it all of the time?

A single coder (the second author) coded all transcripts for the identification and interpretation of key themes, using hierarchical coding achieved through multiple readings and successive codings, supported by keyword analysis (a kind of content analysis) using WMatrix for computer-assisted qualitative discourse analysis [35]. The first author, who had been on site and had personally interacted with participants, reviewed the interpretation for both coherence and representativity; disagreements were resolved, and full consensus was reached.

## Results

### Sample

A total of 21 participants were recruited for the study. Participant demographics are shown in Table 1.

With two exceptions—both of whom said they did some limited walking daily—respondents at all sites admitted in the preintervention interview to doing very little exercise, but said they were concerned about balance and maintaining their health. All thought that group exercises sounded good, agreed to try out telehealth-delivered exercise instructions, and were willing to try a Fitbit tracker although only one or two had seen advertisements for it. In the postintervention interviews, respondents announced they would welcome a second program, liked the group activity, and the telehealth delivery, had little to say about the nursing students, and thought the Fitbit was definitely worth the trouble it took to remember to charge it. Five persons from Garden North were called again after the postintervention call because of their enthusiasm for the program and asked if they would be “ambassadors” to explain the program to other foundation-supported low-income residences; all agreed.

**Table 1:** Participant demographics at Garden Vistas and Garden North (N=21).

Variable		Garden Vistas sample (n=6)	Garden North sample (n=15)
**Gender, n (%)**			
	Female	5 (83)	14 (93)
	Male	1 (17)	1 (7)
Age (years), mean (SD)		72.8 (9.7)	72.3 (7.9)
Age (years), range		58-83	57-85
**Race, n (%)**			
	White	3 (50)	0 (0)
	Black	3 (50)	15 (100)
Ethnicity (not Hispanic or Latino), n (%)		6 (100)	15 (100)
**Marital status, n (%)**			
	Never married	0 (0)	2 (13)
	Married	1 (17)	3 (20)
	Divorced	2 (33)	5 (33)
	Widowed	1 (17)	5 (33)

**Table 2:** Garden Vistas: exercise experience and socialization.

Sample themes and quotations		Code exemplar	Categories
**We can tell you about our exercise but not about ourselves**			
	We’re doing balancing exercises that are helping us a lot, my balance seems to be getting better.	Balance exercise	Exercise types
	The property that we live at we have a class on Monday, Wednesday and Friday where we do core strengthening exercises.	Core exercises for some	“The property that we live at”—not called a home
	We compare our steps: mall walk and garage walk.	Some talk about counting steps	Some conversation
**Lack of socialization contributes to depression**			
	There’s not much socializing, we should repeat the program.	Missing socialization	Lack of socialization
	Maybe the building as a whole can socialize.	Possible solution to no socialization	Lack of socialization
	I fight depression by getting dressed and going anyplace.	Have to fight depression	Depression
	You’re gonna laugh, I make my bed. Then you don’t get back in it.	Make self get up	Depression
	I think it’s just getting together as a group and you know socializing and doing these exercises and having fun doing it together.	What I’d like to see: having fun together	Need more socialization

Garden North differed from Garden Vistas in a number of ways, primarily keyed to residents’ needs and the quality of the community. Although Garden Vistas had more than three times the number of potential participants, only a few agreed to join the project. The major issue seemed to have been their own perceptions of a need for better mental health. As shown in Table 2, Garden Vistas participants had very little to say about the program other than to report on the exercises they had been shown; instead, they wanted to talk about how they tried to combat depression and isolation.

Garden North residents, on the other hand, had a great deal to say—and much of it was about how Garden North was a real community, which accounted, the respondents felt, for their not feeling socially isolated or consistently depressed. Individuals at Garden North had problems—some were former alcoholics or addicts, many had chronic diseases and comorbidities—but they helped one another through the bad times. This is especially noteworthy because the majority of the residents at Garden North, sited in an inner-city urban neighborhood, were of one of the most vulnerable groups in the low-income population, African American [25]. Table 3 displays Garden North’s categories and themes.

**Table 3:** Garden North: exercise experience, recruiting, socialization, and neighborhood.

Sample themes and quotations		Code exemplar	Categories
**Exercise is fun and beneficial**			
	It was fun. It was something I’ve been wanting to do it and after that, I had an opportunity to do it in here. I just went on and then joined in and started doing it and the work got real good.	This is something desired before it started	A person could join in and have fun
	Yeah, I lose a lot. Yeah, it makes me lose a lotta weight. I don’t eat as much as I used to eat so, yeah, everything is fine.	Weight loss is important	Exercise is beneficial
**Recruit older persons individually for success**			
	Everybody start coming by. She started with six people but we ended up with a good group, we have two sections.	Others were curious about the group	Groups expanded quickly
	We’re senior residents up here. And she approached me. I don’t know who she approached here, but she stated what she wanted, and we all complied with that.	Older persons solicited individually to join group	Personalized approach seems to have worked with older people
**We are a community like one of family members**			
	Well, it’s more like a family, a family thing and everybody care about one another over here and everybody come here and they love to be here and they wish they could live in this place. It’s just nice. Everybody gets along real well and we look out for one another, help one another out and all of that and then we go on trips and stuff. It’s just a lotta fun.	We act like a large family, taking care of one another and going out together	We like to do things with one another
	We’re all old. We’re all struggling to make tomorrow or the day-after-tomorrow. Why strong? I guess we’re survivors. We just wanna survive. Some of us are ex-alcoholic, ex-drug-addiction. Well, I guess it just gives you incentive to strive for something better in life.	We’re old, want to help one another survive	Survival means helping one another
**We don’t buy into stereotypes of age**			
	Now, what is old? To me what is old? Old is how you feel. It’s a state of mind. My mother’s 84. She acts like she’s in her 40s or something. I wish I could move as fast as her. She’s just always on the go. She dances. She goes on long drives. She goes out to the beach.	Chronological age doesn’t mean being old	Age is a state of mind
	Is it because we all been here together for so long and we know each other. We know the good, the bad, and the ugly. We always try to help one another out when we’re ill...	We are long-term residents and we know a lot about one another	We know one another very well
**We actively practice socialization**			
	Once we all moved into this building we got to know one another. We used to always have dinners or we’d have breakfast every Saturday morning. We had gotten that started. Then we all joined in together where we had president, vice president, you know, and we put things in paper so we could all see what we wanted to do. Then we’d start decorating the building for Christmas and stuff and Easter.	We started by eating together, then organized, and moved to decorating “our home”	This is a community, not a “property”
**We’re right in the middle of everything here**			
	Yeah, the neighborhood is nice. You’ve got the store. You’ve got the eating place. You’ve got the Chinese, the school, the library, the arcade next door and *[crosstalk]* –And the park and all of that. It’s just nice. It’s a nice neighborhood to be in.	Itemizes what’s available in the immediate neighborhood	Neighborhood has everything a person could want

A year later, when follow-up interviews were conducted with 10 of the 13 remaining Garden North participants, all 10 reported they were continuing to do some exercise if not all the specific exercises that they had learned in the class. Most no longer used or could not find their Fitbits—one complained that she kept forgetting to take it off before washing dishes, another explained that hers “blew up,” but one still wears her watch. All have kept up with some of the walking and the step climbing, all said they would like to take class with one another and liked telehealth. Only one reported that she had become depressed but that visiting a doctor at MUSC and talking about depression with her sister was allowing her to see improvement.

## Discussion

Comments by participants in the two groups index the differences in their attitudinal approaches to the program. Garden Vistas participants noted the lack of socialization in their building and while doing the program. They had little conversation with one another and their interviews typically focused on how to combat depression while living in “the property”: note that nobody called it a residence or a home or even an apartment. It was seen as a property that belongs to somebody or something else, not to them.

Although the program was new at each of the sites, it was easier to administer and to encourage at Garden North because many of the residents felt they lived in a community, with stores, a park, a library, and other places for outside interaction being seen as an easier walk. Interestingly, distance from outside locations was similar at each site. However, automobile and truck traffic patterns at Garden Vistas made walking outside more difficult, with longer isolated stretches between traffic lights and crosswalks and fewer low-income, mom-and-pop stores. In addition, Garden Vistas had little community “feel”; indeed, many residents seemed to be dazed and fearful, seeking isolation while wishing for activities that could involve friendship. Mental health loomed as a much more prevalent issue at Garden Vistas: people at Garden North were aware of one another’s mental health needs, from addiction to depression, in ways that residents at Garden Vistas were not. At the conclusion of the study, interested participants who had been to the majority of the PA sessions were asked if they would be comfortable to continue to lead the sessions twice weekly. Several participants from each site were trained to continue on with the PA groups.

The study had several limitations including a small sample size. The sample was taken from two low-income retirement communities managed by the same property management company, which may limit generalizability to the population; however, the two properties were vastly different in their demographic data. Use of the PA trackers may be limited in this older adult, low-income demographic, although some participants really enjoyed wearing the trackers.

It is clear that PA and socialization is critical to older adults who are aging in place. More importantly, mental health—not just physical health—has to be considered when attempting to engage older adults in group activities. Mental health plays a role in physical health as well as socialization and is a key component to healthy aging in place. Future studies need to be aware of older adults’ mental health needs and these needs should be addressed to encourage socialization and PA. Integrating mindfulness, depression screening, and other mental health screening and education into future aging-in-place interventions is a necessity.

## Abbreviations

MUSC: Medical University of South Carolina
PA: physical activity
PT: physical therapy
